# Improvement of D-Ribose Production from Corn Starch Hydrolysate by a Transketolase-Deficient Strain* Bacillus subtilis* UJS0717

**DOI:** 10.1155/2015/535097

**Published:** 2015-12-02

**Authors:** Zhuan Wei, Jue Zhou, WenJing Sun, FengJie Cui, QinHua Xu, ChangFeng Liu

**Affiliations:** ^1^Hebei Chemical and Pharmaceutical College, Shijiazhuang 050026, China; ^2^School of Food and Biological Engineering, Jiangsu University, Zhenjiang 212013, China; ^3^Shandong Depu Chemical Technology Co., Ltd., Tai'an 271200, China

## Abstract

D-Ribose is a five-carbon sugar and generally used as an energy source to improve athletic performance and the ability. The culture conditions for maximum D-ribose production performance from cheap raw material corn starch hydrolysate were improved by using one-factor-at-a-time experiments and a three-level Box-Behnken factorial design. The optimal fermentation parameters were obtained as 36°C culture temperature, 10% inoculum volume, and 7.0 initial pH. The mathematical model was then developed to show the effect of each medium composition and their interactions on the production of D-ribose and estimated that the optimized D-ribose production performance with the concentration of 62.13 g/L, yield of 0.40 g/g, and volumetric productivity of 0.86 g/L·h could be obtained when the medium compositions were set as 157 g/L glucose, 21 g/L corn steep liquor, 3.2 g/L (NH_4_)_2_SO_4_, 1 g/L yeast extract, 0.05 g/L MnSO_4_·H_2_O, and 20 g/L CaCO_3_. These findings indicated the D-ribose production performance was significantly improved compared to that under original conditions.

## 1. Introduction

D-Ribose (C_5_H_10_O_5_) is a functional five-carbon sugar and plays the important role in life as a ribosyl residue for ATP, RNA, NAD, NADP, FAD, and coenzyme A. D-Ribose has been used to improve athletic performance/ability as an energy source [1] and produce riboflavin (vitamin B_2_), animal feed additives, cosmetics and foods [2], and antiviral and anticancer drugs [3].

Two methods including yeast RNA hydrolysis and chemical synthesis from gluconic acids, glucose, arabinose, and xylose are previously used for preparing D-ribose [4, 5]. For example, D-ribose with a yield of 60–94% was produced by epimerizing D-arabinose in the presence of molybdic and boric acids [4]. However, chemical synthesis processes for D-ribose production suffered from significant disadvantages such as low yield, complex scheme, and recovering/purifying burdens. Currently, almost all the 2000–3000 tonnes of D-ribose produced annually worldwide are obtained by microbial fermentation due to high selectivity, high rate, and high yield of conversion [6]. The microorganisms from genera* Bacillus* are the main D-ribose producers including* B. subtilis* and* B. pumilus*. Most of those strains, however, restricted their usefulness for commercial production due to certain disadvantages such as long fermentation time and lower ribose concentration and productivity. For example, about 40 g/L of D-ribose was produced from 200 g/L of glucose by* Bacillus subtilis* ATCC 21951 after 7-d fermentation [7]. Generally, only* Bacillus* strains deficient in the transketolase and/or D-ribulose-5-phosphate-3-epimerase have the ability to accumulate ribose in the fermented broth since these enzymes will further catalyze the produced ribose to the aromatic amino acids [6]. Our group has focused on the D-ribose fermentation since 2000 and screened several transketolase-deficient* Bacillus* stains with industrial application potency [8–10]. One of the strains,* Bacillus subtilis* B941, was screened to produce 41.8 g/L of D-ribose from 180 g/L of glucose. After mutation with UV irradiation, the mutant strain* Bacillus subtilis* Buvp-24 produced the maximum D-ribose concentration of 55 g/L [9].

Starchy biomass is a promising feedstock for chemical bioproduction due to its abundant availability. Commercial scale for converting starchy biomass to chemicals such as ethanol, lactic acid, and 2-keto-gluconic acid has been realized [11]. Corn starch is an abundant inexpensive renewable resource and larger output in China and the United States [12, 13] while to date the fermentative production of D-ribose from corn starch has not been yet reported.

Statistical techniques have been used for optimizing the culture media to produce microbial metabolites [14–16]. Response surface methodology (RSM), as a collection of statistical techniques for experiment designing, model developing, factors evaluating, and optimum conditions searching, has been extensively applied in optimization of medium composition, conditions of enzymatic hydrolysis, fermentation, and food manufacturing processes [17, 18]. However, very few references with application of statistical techniques to maximize D-ribose production are available. Hence, the objective of the present study was to optimize the process parameters and medium compositions to increase the D-ribose production performance by a transketolase-deficient strain* Bacillus subtilis* UJS0717 using one-factor-at-a-time experiments followed by a three-level Box-Behnken factorial design combining with response surface methodology (RSM).

## 2. Materials and Methods

### 2.1. Bacterial Strain and Media

The D-ribose producer* B. subtilis* UJS0717 was a mutant from Industrial Microbiology Laboratory in Shanxi Institute of Biology and maintained at 4°C in ampoule tube and kept in our laboratory. Stock medium contained 5 g/L of D-sorbitol, 10 g/L of peptone, 2 g/L of NaCl, 2 g of yeast extract, and 20 g/L of agar. A loopful of the stock culture was diluted with sterilized water and inoculated into 20 mL of seed medium containing glucose 20 g/L, yeast extract 3 g/L, K_2_HPO_4_ 3 g/L, and KH_2_PO_4_ 1 g/L, in a 250 mL Erlenmeyer flask, incubated at 36°C, 240 rpm, for 20 h, and used as a seed culture.

Corn starch hydrolysate (CSH) was obtained from Shandong Depu Chemical Technology Co., Ltd. (Taian, Shandong, China), produced by liquefaction and saccharification processes with amylases and glucoamylase. The corn starch hydrolysate contains approximately 30% (w/v) of glucose and 0.6% of protein (total nitrogen × 6.38). CSH was diluted with deionized water to obtain various concentrations of glucose. Other analytical chemicals were obtained from Sigma Aldrich (St. Louis, MO, USA). The basic fermentation medium contained glucose 120 g/L, corn steep liquor 15 g/L, (NH_4_)_2_SO_4_ 7.5 g/L, yeast extract 1 g/L, and MnSO_4_·H_2_O 0.05 g/L. Glucose and other nutrients were sterilized separately at 121°C for 20 min. 20.0 g/L of CaCO_3_ was added to the media for balancing the broth pH.

### 2.2. One-Factor-at-a-Time Experiments

For one-factor-at-a-time experiments investigating effect of glucose, corn steep liquor, and (NH_4_)_2_SO_4_ concentration on D-ribose production, the glucose, corn steep liquor, and (NH_4_)_2_SO_4_ concentrations in the basic fermentation medium were adjusted ranging from 100 g/L to 210 g/L, 5 g/L to 25 g/L, and 1.5 g/L to 9 g/L, respectively. Four fermentation temperatures from 30°C to 39°C, four inoculum volumes from 5% to 20% (v/v), and five initial pH values from 6.0 to 8.0 were used for selecting the optimal temperature, inoculum volume, and initial pH using one-factor-at-a-time experimental design. The fermentation time was 72 hours if not further specified.

### 2.3. Optimization of Fermentation Medium Compositions

To find the interactions and give the precise levels of media compositions including glucose, corn steep liquor, and (NH_4_)_2_SO_4_ significantly influencing D-ribose production, Box-Behnken design for 3 variables at three levels (+1, 0, and −1) was further employed to optimize their concentrations to maximize the D-ribose production by* B. subtilis* UJS0717 based on the one-factor-at-a-time experiments [18]. The statistical matrix included 15 runs of experiments for fitting a second-order response surface.  Table 5 gives the variables, their values, and the experimental design, respectively.

A mathematical model, describing the relationships between the process indices (D-ribose concentration) and the medium component contents in second-order equation, was developed with the least squares method as follows:

(1) Y=β0+β1X1+β2X2+β3X3+β11X12+β22X22+β33X32+β12X1X2+β13X1X3+β23X2X3,

where *Y* is the measured response; *β*_0_ model constant; *X*_1_, *X*_2_, and *X*_3_ are independent variables; *β*_1_, *β*_2_, and *β*_3_ are linear coefficients; *β*_12_, *β*_13_, and *β*_23_ are cross product coefficients; and *β*_11_, *β*_22_, and *β*_33_ are the quadratic coefficients [19]. The accuracy and general ability of the above polynomial model could be evaluated by the coefficient of determination *R* ^2^.

### 2.4. Analytical Methods

The cell growth was represented with dry cell weight (DCW, g/L) by neutralizing the residual CaCO_3_ with 1 M HCl solution, centrifuging at 10000 ×g for 5 min to obtain* B. subtilis* cells, and drying the cells at 80°C to the constant weight.

Glucose and D-ribose were measured using high performance liquid chromatography (Agilent 1100 series, MN, USA) equipped with a SUGAR SH1011 column (Shodex, 8.0 mm ID × 300 mm) and a differential refractometer (Agilent 1100 series). The mobile phase was 0.005 M H_2_SO_4_ at a flow rate of 0.6 mL/min. The column temperature was maintained at 50°C. About 3 mL samples were taken and filtered with Whatman 0.45 *μ*m syringe filter to obtain about 1 mL permeate for HPLC analysis.

The performance of D-ribose production was evaluated based on D-ribose concentration, D-ribose productivity, glucose conversion ratio, and D-ribose yield. D-Ribose productivity was defined as the amount of D-ribose produced per hour liter. D-Ribose yield was calculated by dividing the amount of D-ribose produced by the amount of glucose consumed. All fermentation tests were run in duplicate. Data analysis including analysis of variance was conducted using the SAS System (SAS Institute, Cary, NC, USA).

## 3. Results and Discussion

### 3.1. Effect of Temperature

**Figure 1:**
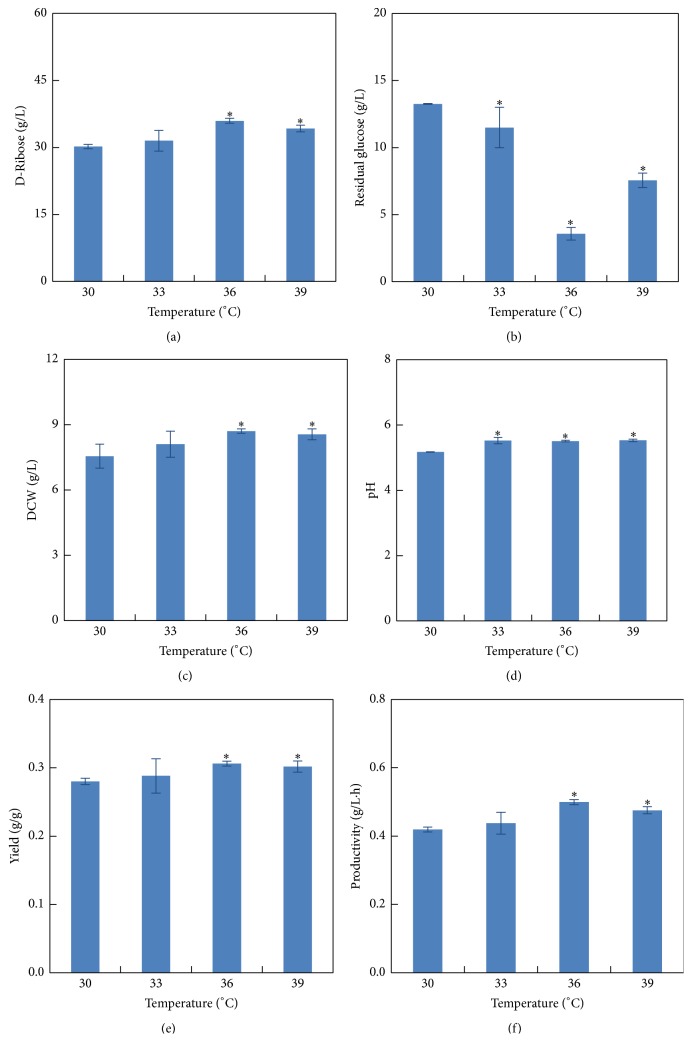
Effect of temperature on D-ribose production performance of* B. subtilis* UJS0717 (fermentation time: 72 h; initial pH 7.0; inoculum volume: 10%, v/v; ^*∗*^ *P* < 0.05 compared to 30°C group).

In order to understand the influence of temperature on the production of D-ribose, fermentation with an initial concentration of 10% (v/v), pH 7.0, and basic fermentation medium was conducted at four temperatures ranging from 30°C to 39°C (Figure 1). D-Ribose productivity was significantly affected by the temperature (*P* < 0.05). The maximum D-ribose productivity of 0.50 g/L·h, glucose utilization ratio of 97.05%, and highest cell concentration of 8.70 g/L were obtained at 36°C after 72 h of fermentation. D-Ribose productivity of 0.44 g/L·h and 0.48 g/L·h and glucose utilization ratio of 90.49% and 93.75% were obtained at 33°C and 39°C, respectively. At 30°C, the ribose productivity and glucose utilization ratio decreased to 0.42 g/L·h and 89.04%, respectively. Kishimoto et al. (1990) found that temperature about 37°C was suitable for D-ribose production by* B. pumilus* NO.716 [23]. Therefore, 36°C appeared to be the optimal temperature for D-ribose production from glucose by* B. subtilis* UJS0717.

### 3.2. Effect of Inoculum Volume

The influence of inoculum volume, ranging from 5 to 20% (v/v), on D-ribose production with conditions of 36°C, pH 7.0, and basic fermentation medium was investigated. As shown in Table 1, D-ribose productivity increased substantially from 0.43 g/L·h to 0.50 g/L·h when inoculum volume increased from 5% to 10% (v/v) (*P* < 0.05). Further increases in inoculum volume (beyond 10%) had no significant effect on the D-ribose production (*P* > 0.05). Similar results were also observed by Ren et al. that high inoculum volume resulted in a negative impact on D-ribose production by* B. subtilis* ptn15-1 [29]. For the present study, an inoculum volume of 10% (v/v) was selected.

### 3.3. Effect of Initial pH

The effect of initial pH, ranging from 6.0 to 8.0, on the performance of D-ribose production by* B. subtilis* UJS0717 was investigated at 36°C, inoculum volume of 10% (v/v) with basic fermentation medium. As shown in Table 2, D-ribose production and cell growth were influenced by the initial pH. D-Ribose productivity increased substantially from 0.43 to 0.50 and glucose utilization ratio increased visibly from 90.78% to 96.31% when initial pH adjusted from 6.0 to 7.0 (*P* < 0.05). Further increases in initial pH (beyond 7.0) had a negative impact on D-ribose production. Park and Seo also found that initial pH about 7.0 was suitable for D-ribose production by* B. subtilis* JY1 [30]. Therefore, pH 7.0 appeared to be optimal for D-ribose production from glucose by* B. subtilis* UJS0717.

### 3.4. Effect of Glucose Concentration

**Figure 2:**
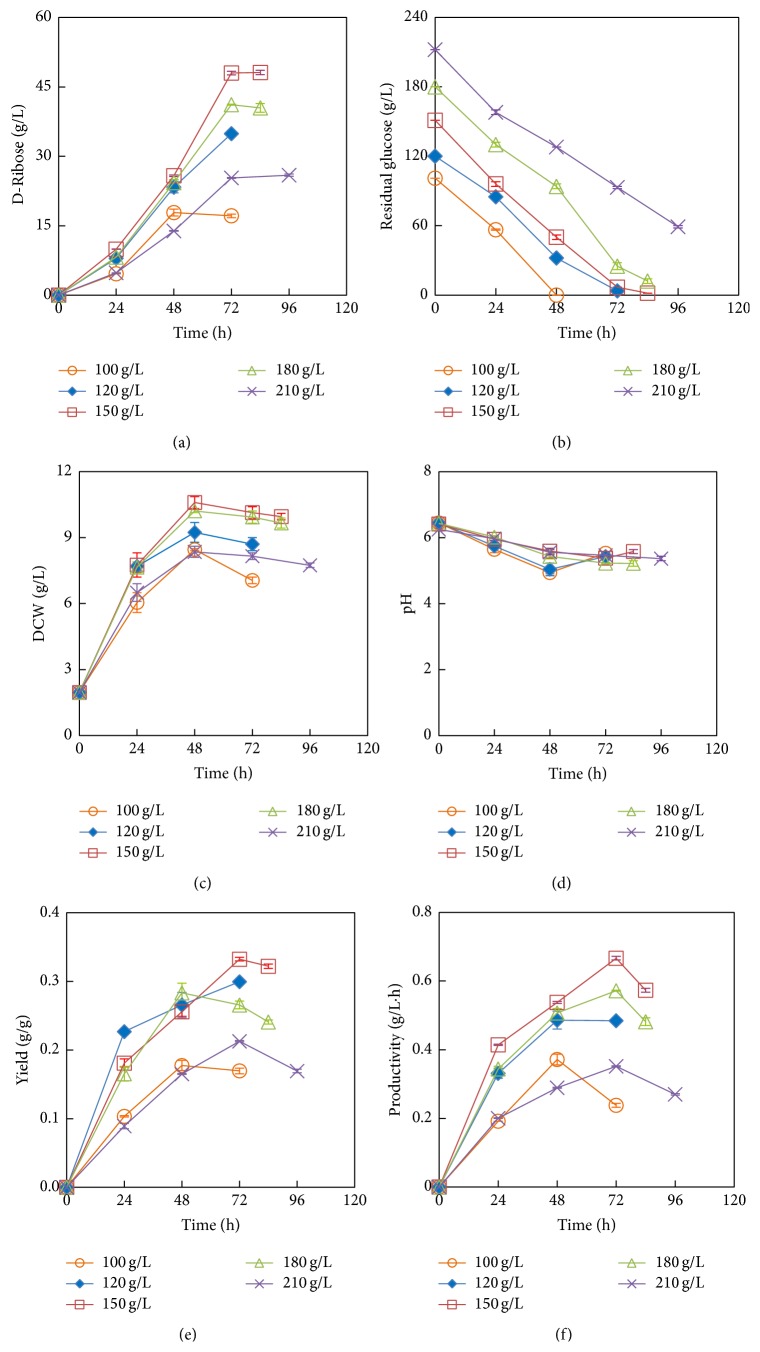
Effect of glucose concentration on D-ribose production performance of* B. subtilis* UJS0717 (corn steep liquor: 15 g/L; (NH_4_)_2_SO_4_: 7.5 g/L).

The effect of glucose concentration ranging from 100 g/L to 210 g/L on the performance of D-ribose production by* B. subtilis* UJS0717 was investigated at 36°C, pH 7.0, and inoculum volume of 10% (v/v). As shown in Figures 2(a) and 2(b), D-ribose concentration increased from 25.81 g/L to 48.01 g/L and glucose concentration decreased gradually from 50.01 g/L to 6.65 g/L with the increase of fermentation time from 48 h to 72 h at glucose concentration of 150 g/L. Within the 48 h fermentation, 100 g/L of glucose was consumed completely. The maximum D-ribose concentration of 48.15 g/L appeared at the initial glucose concentration of 150 g/L. Too high glucose concentrations seemed to possess the inhibition on the glucose consumption and D-ribose production. D-Ribose concentration kept the lower level below 30 g/L and approximately 72% of glucose was consumed during 96 h fermentation when the initial glucose concentration was set as 210 g/L.

Similar trends of cell growth could be observed (Figure 2(c)).* B. subtilis* cells were in the exponential phase with the lower D-ribose production in the 48 h of fermentation and then entered the stationary phase at the 48–96 h with the higher D-ribose production. Glucose concentration of 150 g/L benefited the cell growth and reached maximum concentration of 10.59 g/L.

Figures 2(e) and 2(f) showed the D-ribose yield and productivity of strain* B. subtilis* UJS0717 during the overall fermentation process.* B. subtilis* UJS0717 gave the total D-ribose yield of 0.32 g/g with the medium composed of 150 g/L glucose, followed by 0.30 g/g with 120 g/L and 0.24 g/g with 180 g/L of glucose. High concentrations of glucose (over 180 g/L) had the lower D-ribose yield of 0.17 g/g. The highest volumetric productivity (0.67 g/L·h) was reached at 72 h with the 150 g/L of glucose. With 100, 120, 180, and 210 g/L of glucose, their productivity reached 0.37 g/L·h, 0.49 g/L·h, 0.57 g/L·h, and 0.35 g/L·h at 48 h, 48 h, 72 h, and 72 h, respectively. Therefore, glucose concentration of about 150 g/L appeared to be optimal for D-ribose production by* B. subtilis* UJS0717.

### 3.5. Effect of Corn Steep Liquor Concentration

Corn steep liquor is a major byproduct of the corn wet milling industry and is a low-cost nutrient source available on a large scale [31]. It is the cost effective medium composition due to its high content of nitrogen, water soluble vitamins, amino acids, minerals, and other growth factors [32]. Corn steep liquor as the essential microbial nutrient has been used for production of organic acids, solvents, and enzymes. Previous report also proved that corn steep liquor was an efficient nitrogen source and growth factor for industrial D-ribose fermentation [9, 33]. Herein, to select the optimal corn steep liquor concentration for D-ribose production, five concentrations ranging from 5 g/L to 25 g/L were used. Other culture conditions were 36°C, pH 7.0, inoculum volume of 10% (v/v), and glucose concentration of about 150 g/L. The D-ribose production performances were concluded in Table 3. After 72 h fermentation, cell concentrations increased from 5.71 g/L to 10.71 g/L with the increase of corn steep liquor concentration from 5 g/L to 20 g/L. Lower corn steep liquor concentrations (<15 g/L) resulted in the high residual glucose with the concentration of over 18 g/L and lower D-ribose production of about 28 g/L. With the increase of corn steep liquor concentration to 20 g/L, D-ribose production reached the maximum level of 54.02 g/L with the highest productivity of 0.75 g/L·h and yield of 0.36 g/g. Too high levels of corn steep liquor concentration (25 g/L) seemed to be negative for the D-ribose production (48.84 g/L) and cell growth (10.21 g/L). Therefore, concentration of corn steep liquor of 20 g/L appeared to be optimal for D-ribose production.

### 3.6. Effect of (NH_4_)_2_SO_4_ Concentration

Nitrogen substrates such as (NH_4_)_2_SO_4_ have been shown to be useful for large-scale D-ribose production [25]. In order to select the optimal (NH_4_)_2_SO_4_ concentration on the production of D-ribose, fermentation with 36°C, pH 7.0, inoculum volume of 10% (v/v), glucose concentration of about 150 g/L, and corn steep liquor of 20 g/L was conducted at six (NH_4_)_2_SO_4_ concentrations ranging from 1.5 g/L to 9.0 g/L (Table 4).

D-Ribose productivity was significantly affected by the (NH_4_)_2_SO_4_ concentrations (*P* < 0.05). The maximum D-ribose productivity and yield of 0.84 g/L·h and 0.40 g/g were obtained at (NH_4_)_2_SO_4_ concentration of 3.0 g/L. After 72 h fermentation, cell concentrations increased from 9.40 g/L to 10.77 g/L with the increase of (NH_4_)_2_SO_4_ concentration from 1.5 g/L to 3.0 g/L. Higher (NH_4_)_2_SO_4_ concentrations (>4.5 g/L) resulted in the residual glucose. With (NH_4_)_2_SO_4_ concentration of 6.0 g/L, D-ribose production reached the minimum level of 42.89 g/L with the lowest productivity of 0.60 g/L·h and yield of 0.29 g/g. Too high levels of (NH_4_)_2_SO_4_ concentration (>3.0 g/L) seemed not to benefit the D-ribose production and cell growth, while Srivastava and Wangikar found that 5.0 g/L of (NH_4_)_2_SO_4_ was optimum for D-ribose yield and too high concentrations of (NH_4_)_2_SO_4_ resulted in lower quantities of D-ribose and large quantities of acetic acid and acetoin of 20 g/L and 30 g/L, respectively [34]. Therefore, in our study, concentration of (NH_4_)_2_SO_4_ of 3.0 g/L appeared to be optimal for D-ribose production from glucose by* B. subtilis* UJS0717.

Based on one-factor-at-one-time experimental results, it could be concluded that the optimal culture conditions for D-ribose production from glucose by* B. subtilis* UJS0717 were 36°C, inoculum volume of 10% (v/v), pH 7.0, glucose concentration of 150 g/L, corn steep liquor concentration of 20 g/L, and (NH_4_)_2_SO_4_ concentration of 3.0 g/L. However, one-factor-at-a-time experiments are incapable of reaching the true optimum especially due to interactions among various factors while RSM statistically designs and builds models, evaluates the effects of factors, and searches optimum conditions of factors for the desirable responses. Hence, following response surface methodology combined Box-Behnken design was applied to find the precise levels and interactions among significant factors.

### 3.7. Optimization of Fermentation Media Using Box-Behnken Design (BBD)

In this work, the actual levels of the variables for each of the experiments in the design matrix were calculated and experimental results obtained as given in Table 5.  Table 6 shows the results of the statistical analysis. *F* value of 83.91 and low *P* value (*P* < 0.01) indicated that the model was highly significant and yielded good predictions of the experimental results. The value of the coefficient of determination (*R* ^2^ = 0.9934) also reflected the good fit of the response model [35], which showed that 99.34% of the sample variation in the experiments was explained by the independent variables. The coefficient of variation (CV = 2.02% < 10%) indicated the experiment was accurate and reliable. The value of 0.0556 for lack of fit implies that it is not significant comparing to the pure error and that the model equation was adequate for predicting D-ribose concentration. All the above analytical results demonstrated that the model for D-ribose concentration was appropriate in terms of the Box-Behnken design.

The following second-order polynomial equation based on the multiple regression analysis explained the relationship between variables and D-ribose concentration:

(2) Y=60.29+5.09X1+0.93X2+1.42X3−11.52X12−3.42X22−4.00X32−0.24X1X2−0.19X1X3−1.15X2X3,

where *Y* stands for the response variable (D-ribose concentration) and *X*_1_, *X*_2_, and *X*_3_ are the actual values of D-glucose, corn steep liquor, and (NH_4_)_2_SO_4_ concentrations, respectively. In this equation, the signs of the linear coefficients of *X*_1_, *X*_2_, and *X*_3_ were positive. This result indicated that glucose, corn steep liquor, and (NH_4_)_2_SO_4_ had a synergistic effect on the production of D-ribose. The signs of the coefficients of *X*_1_ *X*_2_, *X*_1_ *X*_3_, *X*_2_ *X*_3_, *X*_1_^2^,  *X*_2_^2^, and *X*_3_^2^ were negative, indicating that they had an inverse effect on D-ribose concentration [35]. The first-order and the quadratic main effects of glucose concentration were highly significant (*P* < 0.01) according to the *P* value of the model. This result indicated that the factor had significant effect on the production of D-ribose. Therefore, the different ratio of the factor in fermentation would affect the production of D-ribose, and small variations in the factor would produce large changes in the results.

**Figure 3:**
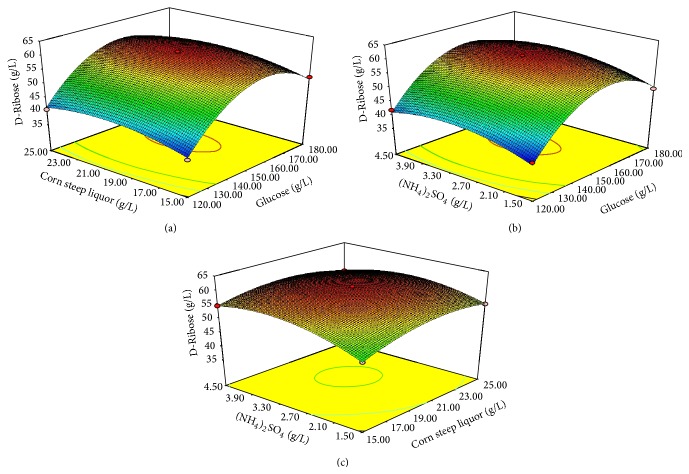
(a) The 3D-plot and 2D-projection showing the interaction between glucose and corn steep liquor at 3.0 g/L (NH_4_)_2_SO_4_ on D-ribose concentration (*Y*). (b) The 3D-plot and 2D-projection showing the interaction between glucose and (NH_4_)_2_SO_4_ at 20 g/L corn steep liquor on D-ribose concentration (*Y*). (c) The 3D-plot and 2D-projection showing the interaction between corn steep liquor and (NH_4_)_2_SO_4_ at 150 g/L glucose on D-ribose concentration (*Y*).

The three-dimensional (3D) response surface plots drawn by Design-Expert 8.0.6.1 software based on the model equations were used to explain the interactions among the variables and to determine the optimal ratios of each component for the production of D-ribose. The response surface shapes reflected the nature and range of different components, and the peaks suggested that the optimum points were within the design limits. In this experiment, each plot was generated for the interactive effects of two variables on the production of D-ribose while holding the other factor at “zero” levels.  Figure 3 was the 3D-surface plot and 2D-projection.

The maximum D-ribose concentration of 61.01 g/L was obtained by solving the model regression equation with the medium composed of 157 g/L glucose, 21 g/L corn steep liquor, and 3.2 g/L (NH_4_)_2_SO_4_. To confirm the predicted results and verify the model, the above-calculated critical levels of the three variables were used to produce D-ribose, and the mean value of D-ribose production was 62.13 ± 1.16 g/L, which was in agreement with the predicted value (61.01 g/L).

**Figure 4:**
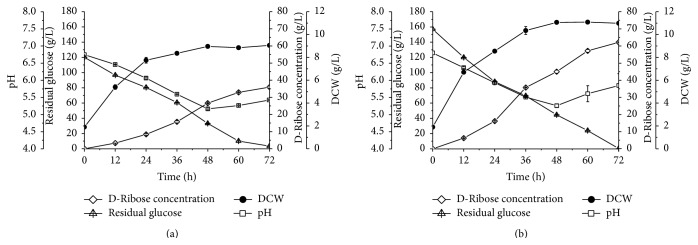
Comparison of D-ribose production performance of* B. subtilis* UJS0717 before and after optimization ((a) before optimization; (b) after optimization).

Figure 4 compared the residual glucose concentration, pH, cell concentration, and D-ribose concentration before and after optimization. Cell concentration reached 9.05 g/L during 72 h cultivation, and relative lower D-ribose concentration of 35.95 g/L, yield of 0.31 g/g, and productivity of 0.50 g/L·h were finally obtained under the original fermentation conditions (Figure 4(a)). After optimization, the substrate glucose was utilized completely and cell concentration was achieved at 10.99 g/L after 72 h cultivation. D-Ribose concentration, yield, and productivity reached 62.13 g/L, 0.40 g/g, and 0.86 g/L·h by* B. subtilis* UJS0717, which were higher than those under the original conditions (Figure 4(b)).

Genus* Bacillus* is the main D-ribose producer which utilizes glucose, gluconic acid, or xylose as substrate.  Table 7 presented the D-ribose producing strains and their fermentation performance from this work and from literature reports. Most D-ribose producing strains in Table 7 had the capacity to produce D-ribose over 60 g/L. The strain* B. subtilis* UJS0717 used in this work is a comparable D-ribose producing bacterium that is able to produce D-ribose with the concentration of 62.13 g/L from 157 g/L glucose and high efficiency of converting glucose to D-ribose.

## 4. Conclusion

The transketolase-deficient strain* B. subtilis* UJS0717 produced D-ribose at level of 62.13 g/L with yield of 0.40 g/g and volumetric productivity of 0.86 g/L·h, which was therefore potentially useful as an industrial D-ribose producer from cheap raw material corn starch hydrolysate. Semicontinuous/continuous fermentation and scale-up experiments for D-ribose production are ongoing in our lab for further improving the D-ribose production performance and evaluating the technical feasibility.

## Figures and Tables

**Table 1:** Effect of inoculum volume on D-ribose production performance by *B. subtilis* UJS0717.

Inoculum volume (v/v)	Initial glucose (g/L)	Residual glucose (g/L)	Glucose consumption rate (%)	Glucose consumption rate (g/L·h)	Cell concentration (g/L)	D-Ribose (g/L)	D-Ribose yield (g/g)	D-Ribose productivity (g/L·h)
5%	122.0	12.75 ± 0.14	89.55 ± 0.11	1.52 ± 0.00	7.79 ± 0.01	31.23 ± 0.35	0.29 ± 0.00	0.43 ± 0.00
10%	122.0	4.50 ± 0.35^*∗*^	96.31 ± 0.29^*∗*^	1.63 ± 0.00^*∗*^	8.70 ± 0.32^*∗*^	36.00 ± 0.75^*∗*^	0.31 ± 0.00^*∗*^	0.50 ± 0.00^*∗*^
15%	122.0	6.53 ± 0.58^*∗*^	94.65 ± 0.47^*∗*^	1.60 ± 0.00^*∗*^	9.30 ± 0.20^*∗*^	34.73 ± 0.66^*∗*^	0.30 ± 0.00^*∗*^	0.48 ± 0.00^*∗*^
20%	122.0	6.51 ± 0.67^*∗*^	94.66 ± 0.55^*∗*^	1.60 ± 0.00^*∗*^	9.37 ± 0.10^*∗*^	34.91 ± 0.58^*∗*^	0.30 ± 0.00^*∗*^	0.49 ± 0.00^*∗*^

^*∗*^ *P* < 0.05 compared to 5% inoculum volume (v/v) group.

**Table 2:** Effect of the initial pH on D-ribose production performance by *B. subtilis* UJS0717.

Initial pH	Initial glucose (g/L)	Residual glucose (g/L)	Glucose consumption rate (%)	Glucose consumption rate (g/L·h)	Cell concentration (g/L)	D-Ribose (g/L)	D-Ribose yield (g/g)	D-Ribose productivity (g/L·h)
6.0	122.0	11.25 ± 0.12	90.78 ± 0.10	1.54 ± 0.00	7.95 ± 0.15	30.92 ± 0.47	0.28 ± 0.00	0.43 ± 0.00
6.5	122.0	8.95 ± 0.05^*∗*^	92.66 ± 0.04^*∗*^	1.57 ± 0.00^*∗*^	8.60 ± 0.25^*∗*^	31.35 ± 0.35	0.28 ± 0.00	0.43 ± 0.00
7.0	122.0	4.50 ± 0.35^*∗*^	96.31 ± 0.29^*∗*^	1.63 ± 0.00^*∗*^	8.70 ± 0.32^*∗*^	36.00 ± 0.75^*∗*^	0.31 ± 0.00^*∗*^	0.50 ± 0.00^*∗*^
7.5	122.0	6.85 ± 0.18^*∗*^	94.39 ± 0.15^*∗*^	1.60 ± 0.00^*∗*^	8.35 ± 0.35^*∗*^	34.57 ± 0.55^*∗*^	0.30 ± 0.00^*∗*^	0.48 ± 0.01^*∗*^
8.0	122.0	8.55 ± 0.14^*∗*^	92.99 ± 0.11^*∗*^	1.57 ± 0.00^*∗*^	8.15 ± 0.25^*∗*^	31.76 ± 0.68	0.28 ± 0.00	0.44 ± 0.00^*∗*^

^*∗*^ *P* < 0.05 compared to pH 6.0 group.

**Table 3:** Effect of corn steep liquor concentration on D-ribose production performance by *B. subtilis* UJS0717.

Corn steep liquor concentration (g/L)	Initial glucose (g/L)	Residual glucose (g/L)	Glucose consumption rate (%)	Glucose consumption rate (g/L·h)	Cell concentration (g/L)	D-Ribose (g/L)	D-Ribose yield (g/g)	D-Ribose productivity (g/L·h)
5	152.0	28.00 ± 1.14	81.58 ± 0.93	1.72 ± 0.02	5.71 ± 0.11	28.89 ± 0.94	0.23 ± 0.01	0.40 ± 0.00
10	152.0	18.00 ± 0.14^*∗*^	88.16 ± 0.09^*∗*^	1.86 ± 0.00^*∗*^	7.41 ± 0.10^*∗*^	34.15 ± 1.54^*∗*^	0.25 ± 0.01^*∗*^	0.47 ± 0.00^*∗*^
15	152.0	6.40 ± 0.06^*∗*^	95.79 ± 0.04^*∗*^	2.02 ± 0.00^*∗*^	10.16 ± 0.16^*∗*^	48.25 ± 0.24^*∗*^	0.33 ± 0.00^*∗*^	0.67 ± 0.00^*∗*^
20	152.0	0.13 ± 0.04^*∗*^	99.91 ± 0.03^*∗*^	2.11 ± 0.00^*∗*^	10.71 ± 0.21^*∗*^	54.02 ± 0.89^*∗*^	0.36 ± 0.00^*∗*^	0.75 ± 0.00^*∗*^
25	152.0	0.18 ± 0.04^*∗*^	99.88 ± 0.03^*∗*^	2.11 ± 0.00^*∗*^	10.21 ± 0.09^*∗*^	48.84 ± 0.26^*∗*^	0.32 ± 0.00^*∗*^	0.68 ± 0.00^*∗*^

^*∗*^ *P* < 0.05 compared to corn steep liquor concentration of 5 g/L group.

**Table 4:** Effect of (NH_4_)_2_SO_4_ concentration on D-ribose production performance by *B. subtilis* UJS0717.

(NH_4_)_2_SO_4_ concentration (g/L)	Initial glucose (g/L)	Residual glucose (g/L)	Glucose consumption rate (%)	Glucose consumption rate (g/L·h)	Cell concentration (g/L)	D-Ribose (g/L)	D-Ribose yield (g/g)	D-Ribose productivity (g/L·h)
1.5	151.0	3.85 ± 0.14	97.45 ± 0.09	2.04 ± 0.00	9.40 ± 0.30	45.81 ± 1.23	0.31 ± 0.01	0.64 ± 0.01
3.0	151.0	0.12 ± 0.01^*∗*^	99.92 ± 0.00^*∗*^	2.09 ± 0.00^*∗*^	10.77 ± 0.17^*∗*^	60.11 ± 0.12^*∗*^	0.40 ± 0.00^*∗*^	0.84 ± 0.01^*∗*^
4.5	151.0	0.29 ± 0.01^*∗*^	99.81 ± 0.01^*∗*^	2.09 ± 0.00^*∗*^	10.65 ± 0.15^*∗*^	56.25 ± 0.67^*∗*^	0.37 ± 0.00^*∗*^	0.78 ± 0.01^*∗*^
6.0	151.0	5.59 ± 0.21^*∗*^	96.30 ± 0.14^*∗*^	2.02 ± 0.00^*∗*^	9.15 ± 0.15	42.89 ± 0.17^*∗*^	0.29 ± 0.00^*∗*^	0.60 ± 0.00^*∗*^
7.5	151.0	0.80 ± 0.14^*∗*^	99.47 ± 0.09^*∗*^	2.09 ± 0.00^*∗*^	10.55 ± 0.35^*∗*^	54.56 ± 0.30^*∗*^	0.36 ± 0.00^*∗*^	0.76 ± 0.00^*∗*^
9.0	151.0	5.43 ± 0.04^*∗*^	96.40 ± 0.28^*∗*^	2.02 ± 0.00^*∗*^	9.25 ± 0.25	43.04 ± 0.64^*∗*^	0.30 ± 0.00^*∗*^	0.60 ± 0.00^*∗*^

^*∗*^ *P* < 0.05 compared to (NH_4_)_2_SO_4_ concentration of 1.5 g/L group.

**Table 5:** Box-Behnken design matrix along with the experimental and predicted values.

Run	Actual and coded level of variables			D-Ribose production (g/L)	
	*X*_1_ (g/L)	*X*_2_ (g/L)	*X*_3_ (g/L)	Experimental	Predicted
1	150 (0)	20 (0)	3.0 (0)	60.64 ± 1.34	60.29
2	150 (0)	25 (+1)	4.5 (+1)	54.32 ± 2.12	54.25
3	180 (+1)	25 (+1)	3.0 (0)	52.09 ± 1.22	51.31
4	150 (0)	15 (−1)	1.5 (−1)	49.49 ± 2.01	49.56
5	150 (0)	20 (0)	3.0 (0)	60.20 ± 1.67	60.29
6	180 (+1)	20 (0)	4.5 (+1)	50.23 ± 1.34	51.08
7	120 (−1)	25 (+1)	3.0 (0)	40.82 ± 3.10	41.61
8	120 (−1)	20 (0)	4.5 (+1)	42.00 ± 3.12	41.28
9	180 (+1)	20 (0)	1.5 (−1)	47.92 ± 1.45	48.64
10	180 (+1)	15 (−1)	3.0 (0)	50.72 ± 1.33	49.93
11	150 (0)	20 (0)	3.0 (0)	60.04 ± 2.03	60.29
12	150 (0)	25 (+1)	1.5 (−1)	53.64 ± 1.34	53.71
13	120 (−1)	20 (0)	1.5 (−1)	38.92 ± 1.43	38.07
14	120 (−1)	15 (−1)	3.0 (0)	38.48 ± 1.98	39.26
15	150 (0)	15 (−1)	4.5 (+1)	54.75 ± 2.50	54.68

**Table 6:** Analysis of variance for the response surface quadratic model of D-ribose concentration of Box-Behnken design.

Source	Sum of squares	df	Mean square	*F* value	*P* value Prob. > *F*
Model	779.43	9	86.60	83.91	<0.0001^*∗∗*^
*X*_1_	207.47	1	207.47	201.01	<0.0001^*∗∗*^
*X*_2_	6.90	1	6.90	6.69	0.0491^*∗*^
*X*_3_	16.05	1	16.05	15.55	0.0109^*∗*^
*AB*	0.24	1	0.24	0.23	0.6532
*AC*	0.15	1	0.15	0.14	0.7203
*BC*	5.24	1	5.24	5.08	0.0739
*A* ^2^	490.36	1	490.36	475.09	<0.0001^*∗∗*^
*B* ^2^	38.80	1	38.80	37.59	0.0017^*∗∗*^
*C* ^2^	59.13	1	59.13	57.29	0.0006^*∗∗*^
Residual	5.16	5	1.03		
*Lack of fit*	4.97	3	1.66	17.15	0.0556
*Pure error*	0.19	2	0.097		
Cor. total	784.59	14			

Note: CV% = 2.02; *R* ^2^ = 0.9934; Adj. *R* ^2^ = 0.9816; Pred. *R* ^2^ = 0.8981. Note: *X*_1_ = glucose (g/L); *X*_2_ = corn steep liquor (g/L); *X*_3_ = (NH_4_)_2_SO_4_ (g/L).

^*∗*^ *P* < 0.05.

^*∗∗*^ *P* < 0.01.

**Table 7:** Summarized results of D-ribose biosynthesis previously described in literature.

Strain	Culture mode	Glucose (g/L)	D-Ribose (g/L)	Fermentation time (h)	Reference
*B. subtilis* UJS0717	Batch	157	62.13	72	In the present work
*B. pumilus* ATCC 21357	Batch	125	31	55	[20]
*B. subtilis *ATCC 31092	Batch	150	67	60	[21]
*Bacillus *sp. EMP-58	Batch	140	64	55	[22]
*B. subtilis *ATCC 21951	Batch	200	95	72	[23]
*B. subtilis* IFO 1538	Batch	160	62	72	[24]
*B. subtilis *ATCC 21951	Batch	100/100^a^	60	110	[25]
*B. subtilis *ATCC 21951	Batch	100/50^b^	45	84	[8]
*B. subtilis *C1-B941	Batch	180	60.9	68	[9]
*B. subtilis *SPK1	Fed-batch	20/20 + 200/50^c^	46.6	63	[2]
*B. subtilis *EC2	Batch	200	83.4	42	[26]
*B. subtilis *NJT-1507	Shake-flask	172.75	88.57	72	[27]
*B. subtilis *NJT-1507	Batch	172.75	95.27	72	[27]
*B. subtilis *XB02	Single-stage, continuous	200^d^	68.7	160	[28]

^a^100 g/L glucose plus 100 g/L D-gluconic acid.

^b^100 g/L glucose plus 50 g/L D-gluconic acid.

^c^After initial sugars of 20 g/L xylose and 20 g/L glucose were consumed completely, a sugar mixture of 200 g/L xylose and 50 g/L glucose was fed stepwise into a bioreactor.

^d^Initial glucose 200 g/L, starting time 24 h, dilution rates 0.006/h, and influent glucose concentration 200 g/L.
